# Repurposing Osimertinib and Gedatolisib for Glioblastoma Treatment: Evidence of Synergistic Effects in an In Vitro Phenotypic Study

**DOI:** 10.3390/ph17121623

**Published:** 2024-12-03

**Authors:** Vitória Santório de São José, Bruno Marques Vieira, Vivaldo Moura Neto, Lidia M. Lima

**Affiliations:** 1 Laboratório de Avaliação e Síntese de Substâncias Bioativas (LASSBio®), Instituto Nacional de Ciência e Tecnologia de Fármacos e Medicamentos (INCT-INOFAR), Universidade Federal do Rio de Janeiro, Rio de Janeiro 21941-902, Brazil; vitoriassjose@gmail.com; 2 Programa de Pós-Graduação em Farmacologia e Química Medicinal, Instituto de Ciências Biomédicas, Universidade Federal do Rio de Janeiro, Rio de Janeiro 21941-902, Brazil; 3 Laboratório de Biomedicina do Cérebro, Instituto Estadual do Cérebro Paulo Niemeyer (IECPN), Rio de Janeiro 20231-092, Brazil; brunomarquesv@gmail.com (B.M.V.); vivaldomouraneto@gmail.com (V.M.N.); 4 Laboratório de Medicina Experimental e Saúde, Instituto Oswaldo Cruz, Fiocruz, Rio de Janeiro 21040-900, Brazil

**Keywords:** glioblastoma, EGFR, PI3K, mTOR, synergism, Osimertinib, Gedatolisib

## Abstract

**Background/Objectives:** Glioblastoma is a malignant tumor with a poor prognosis for the patient due to its high lethality and limited chemotherapy available. Therefore, from the point of view of chemotherapy treatment, glioblastoma can be considered an unmet medical need. This has led to the investigation of new drugs for monotherapy or associations, acting by synergistic pharmacological mechanisms. **Methods:** Here, we propose the combination of Osimertinib (a potent EGFR inhibitor) and Gedatolisib (a potent PI3K/mTOR dual inhibitor) through an in vitro phenotypic study using five human GB lines and establish the cytotoxic potency, selectivity, and effect on proliferation, apoptosis, and cell cycle by simultaneously inhibiting EGFR, PI3K, and mTOR. **Results:** Cytotoxic potency of Gedatolisib and Osimertinib in the selected GB cell lines was determined, which highlighted the synergistic response from their combination and its impact on migration reduction, G0/G1 cell cycle arrest, GB cytotoxicity, and apoptosis-inducing effects for different GB cell lines. **Conclusions:** From the drug combination studies in phenotypic in vitro models, it was possible to suggest a new potential treatment for glioblastoma that justifies further safe in vivo phases of preclinical trials with the combination.

## 1. Introduction

Glioblastoma (GB) is a high-grade glioma that arises from the uncontrolled proliferation of glial cells. GB is an orphan disease with a survival rate of 9–15 months after diagnosis. Currently, the standard treatment is based on surgical resection of the affected area, when possible, along with radiotherapy and chemotherapy using DNA alkylating agents such as temozolomide (TMZ). TMZ is a prodrug that will lead to the formation of the active metabolite methyl-diazonium ion. Resistance to TMZ is an essential medical problem in treating GB [1]. The expression of O6-methylguanine-DNA methyl transferase (MGMT) is considered one of the most common mechanisms of resistance to TMZ. Therefore, GB can be viewed as an unmet medical need, and several researchers have sought to identify new therapeutic alternatives for its treatment, including drug repurpose studies [2,3], which must be based on prior knowledge of the pathophysiology of the target disease. In approximately 80% of cases, GB cells’ uncontrolled proliferation is due to alterations in the phosphoinositide 3-kinase (PI3K) signaling pathway. Genetic modifications observed in GB cells include mutations in epidermal growth factor receptor (EGFR), phosphatase and tensin homolog (PTEN), p53, and cyclin-dependent kinase inhibitor 2A/B [4]. This genetic dysregulation leads to excessive proliferation, inhibition of apoptosis, and increased invasive capacity. Consequently, inhibitors of PI3K/mammalian target of rapamycin (mTOR) are potential candidates for developing novel GB treatments. Gedatolisib (also named PKI-587) is a dual PI3K/mTOR inhibitor developed by Pfizer and is currently in clinical trials for treating several tumors [5]. Another critical target in cancer is the EGFR, a tyrosine kinase responsible for cell proliferation and survival processes. This molecule is overexpressed in 40–90% of GBs [6]. In 20% of the cases, EGFR presents a mutation (EGFRvIII) that constitutively activates the PI3K/protein kinase B (Akt)/mTOR pathway, making EGFR inhibitors an alternative therapy to control or treat the GB [7,8]. Osimertinib (Osi) is an irreversible inhibitor of EGFR with good BBB permeability that has been studied as a possible alternative for GB treatment [9]. In this paper, we propose the repurposing of Osi and Gedatolisib (PKI-587) for GB treatment, comparing monotherapies with their combination based on the evaluation of their cytotoxic potency, synergistic effect, and mechanism of action in a phenotypic model using different GB cell lines.

## 2. Results

### 2.1. Temozolomide Resistance Mechanisms and EGFRvIII/PI3Kβ/Vimentin Expression in GB Cell Lines

**Figure 1:**
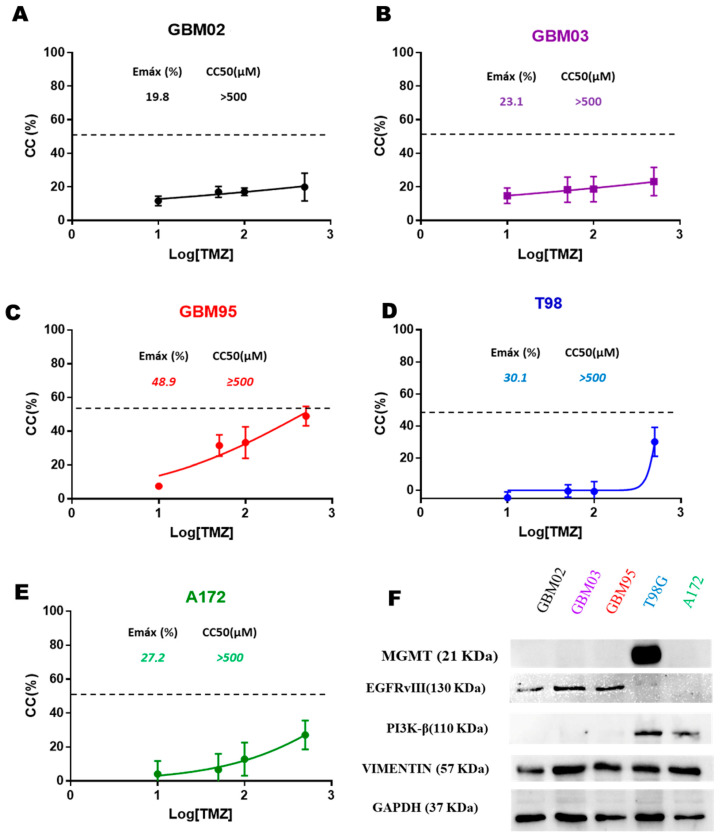
GB cell lines are resistant to TMZ. GBM02 (**A**), GBM03 (**B**), GBM95 (**C**), T98G (**D**), and A172 (**E**) cell lines were treated with different concentrations of TMZ for 72 h. Untreated cells (**F**) MGMT, EGFRvIII, PI3K p110β, and Vimentin protein expressions were measured via Western blot. Values are means ± SEM of three independent experiments in triplicate.

In vitro TMZ effects on cell viability were evaluated using an MTT assay to assess the concentration of cytotoxicity of 50% (CC_50_), which is the concentration that reduces the number of viable cells by 50%, compared with the control. A CC_50_ less than 500 μM means high potency. As shown in Figure 1, the GB cell lines GBM02 (A), GBM03 (B), GBM95 (C), T98G (D), and A172 (E) were resistant to the TMZ treatment, showing low potency (CC_50_ higher than 500 μM) and efficacy (19.8%, 23.1%, 48.9%, 30.1%, and 27.2%, respectively). This CC_50_ in all the GB lineages shows a limited potency against GB in vitro. We performed a western blot analysis to unveil potential TMZ mechanisms and seek novel therapeutical strategies. As shown in Figure 1F and Supplementary Figure S1, we identified the EGFRvIII mutation in the clinical isolates GBM02, GBM03, and GBM95. Furthermore, the PI3K p110β expression was identified in the T98G and A172 lineages. Vimentin, a poor prognostic factor, was expressed in all GB cell lines, indicating high invasion capacity.

### 2.2. Osimertinib and PKI-587 Are Potent and Selective GB Inhibitors

Considering the relevance of EGFRvIII and PI3K p110β inhibition as a possibility to treat GB, we propose the simultaneous triple-inhibition of EGFR (both wt and EGFRvIII), PI3K, and mTOR. First, the cytotoxic potency of Osi and PKI-587 was determined against GBM02, GBM03, GBM95, T98G, and A172. Osi (EGFR inhibitor) displayed a potent effect in all cell lines tested in the MTT assay, displaying a CC_50_ ranging, at 72 h, from 6.6 to 13.01 μM for all GB cell lines studied and a maximum cytotoxic response (Emax) of over 97% (Table 1). At 24 h, CC_50_ values varied between 9.9 μM (T98G) and 29 μM (A172), with a maximum response of 99% for all GB cell lines, except for T98G (Emax of 97.9%, Table 1). Regarding the PKI (Gedatolisib) cytotoxic potency on the GBM02, GBM03, GBM 95, T98G, and A172 cell lines, it displayed time-dependent cytotoxicity against all GB cell lines, with potency varying from 0.03 μM to 0.55 μM (72 h MTT). In the 24 h MTT test, PKI-587 was less cytotoxic, with CC_50_ ranging from 2.1 μM to 54.2 μM and Emax from 57.7 to 98.4% (Table 1) (Supplementary Figures S3 and S4). To evaluate the cytotoxic selectivity index (SI), which reflects the extent to which a given compound is cytotoxic to tumor cells as opposed to non-tumor cells, Osi and PKI-587 were studied against hASTR from tumor-free patients using the 72 h MTT test. As demonstrated in Table 1, Osi exhibited an SI ranging from 1 to 2. The SI for PKI ranged between 51 and 933.

### 2.3. Synergistic Effect and Selectivity Improvement of Osimertinib and PKI-587 Combination

**Figure 2:**
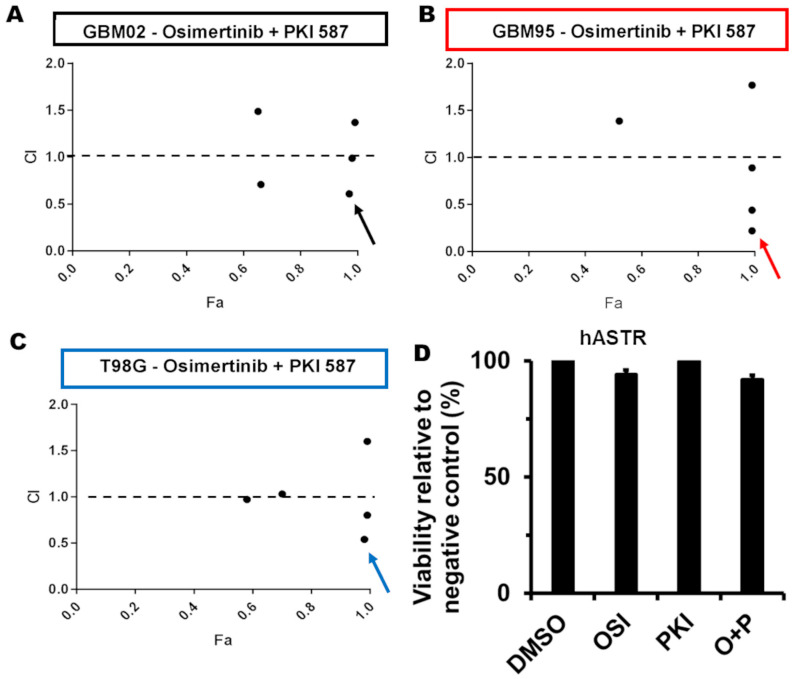
Synergistic effect of triple EGFR/PI3K/mTOR inhibition. Data show GBM02 (**A**), GBM95 (**B**), and T98G (**C**) fraction affected (Fa) versus combination index (CI) plots. The CI values were calculated using CompuSyn version 1.0. The best combination score was indicated by a black arrow (GBM02), a red arrow (GBM95), or a blue arrow (T98G). (**D**) shows the viability of human astrocytes (hASTR) incubated with 5.5 μM Osimertinib, 0.0155 μM PKI-587 (the combination represented by the red arrow in (**B**), isolated or in combination. Values are means ± SEM of three independent experiments in triplicate.

The potential synergism from the combination of Osi and PKI-587 was studied using the Chou–Talalay method [10]. According to the methodology, the synergistic effect is observed when the combination of drug A (Osi) with drug B (PKI-587) results in a combination index value of less than 1. In Figure 2, we see the synergistic effect of the combinations on GBM02 (A), GBM95 (B), and T98G (C) cell lines at 72 h. The best combination, 5.5 μM of Osi and 0.0155 μM of PKI-587 (red arrow, Figure 2B), leads to a viability loss of 92% for GBM95. On the other hand, as demonstrated in Figure 2D, no reduction in hASTR viability was observed using 5.5 μM of Osi and 0.0155 μM of PKI-587 in monotherapy or association. For a clear result, the summarized killing for those concentrations is as follows: GBM95—5.5 μM of Osi reduced 80% of cell viability, 0.0155 μM of PKI-587 reduced 63%, and the combination reduced 92%; hASTR—5.5 μM of Osi reduced 6% of the cell viability, 0.0155 μM of PKI-587 reduced 2%, and the combination reduced 8%. GBM02—13 μM of Osi reduced 35% of cell viability, 0.093 μM of PKI-587 reduced 37%, and the combination reduced 98%; T98G—13 μM of Osi reduced 44% of cell viability, 0.186 μM of PKI-587 reduced 41%, and the combination reduced 98%.

Those initial in vitro results indicate that simultaneous EGFR/PI3K/mTOR inhibition is potentially a safer and more effective strategy for GB therapy.

### 2.4. The Combination Reduces Migration, Leading GB Cell Lines to G0/G1 Arrest In Vitro

**Figure 3:**
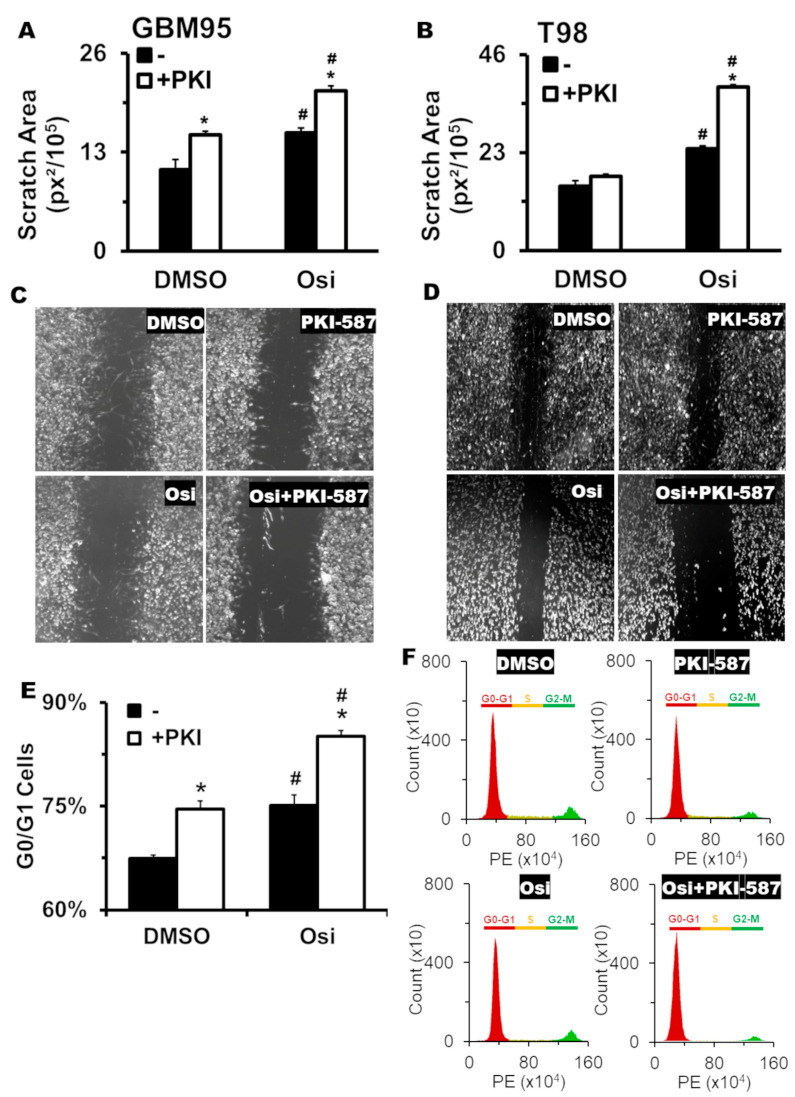
GB treatment impairs the migration and cell cycle. GBM95 (**A**,**C**) and T98G (**B**,**D**) cells were treated with Osi (second closed bar), PKI-587 (first open bar), or the combination (second open bar) in a scratch wound assay. The scratch area (**A**,**B**) was measured using photomicrography ((**C**,**D**) 200× magnification), obtained 24 h after the scratch. GBM95 cell cycle (**E**,**F**) was measured by PI incorporation in flow cytometry (**F**), and the 2n population was set as G1/G0 phase (**E**). In all cases, the “DMSO” group was administered with PBS + 2%DMSO. #, significant differences between the indicated group and the “DMSO” group (*p* < 0.05) in ANOVA. *, significant differences between the indicated group and the “-PKI” respective group (*p* < 0.05) in ANOVA. n = 4 for all groups. (**C**,**D**,**F**) display a representative analysis.

Next, we evaluate the effectiveness of the combination of Osi and PKI-587 (triple inhibition of EGFR/PI3K/mTOR) in the migration response of GB cells using the scratch wound assay. As shown in Figure 3A–D, the Osi and PKI-587 alone (for GBM95) or in combination (for GBM95 and T98G) significantly reduced cell migration. The combination, however, showed increased inhibition compared to monotherapy. For the T98G cell line (Figure 3B,D), PKI-587 monotherapy did not show a statistical difference from the DMSO control group.

Further, we analyzed the cell cycle profile after therapy. As shown in Figure 3E,F, GBM95 cells displayed a G0/G1 arrest in Osi and PKI-587 monotherapies and improved in the combination.

### 2.5. The Combination of Osimertinib and PKI-587 Shows a Cytotoxic Profile and Induces In Vitro Apoptosis

**Figure 4:**
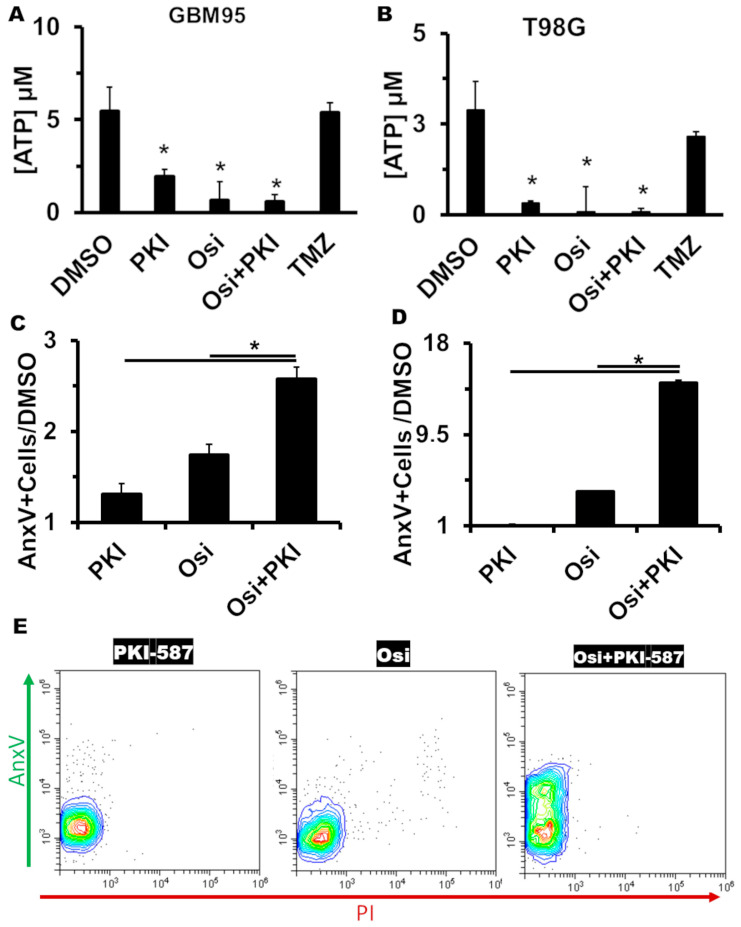
GB treatment induces apoptosis. GBM95 (**A**,**C**,**E**) and T98G (**B**,**D**) cells were treated with Osi, PKI-587, or the combination. In (**A**,**B**), the mono- and polytherapy reduced ATP levels, showing a cytotoxic effect, while the TMZ treatment did not decrease the ATP levels. Compared to monotherapies, polytherapy increased the annexin V for GBM95 (**C**,**E**) and T98G (**D**). *, significant differences between the indicated group and the “DMSO” group (**A**,**B**; *p* < 0.05) in ANOVA, or significant differences between the indicated groups (**C**,**D**; *p* < 0.05) in ANOVA. Values are means ± SEM of two independent experiments in triplicate (**A**,**B**). n = 4 for all groups. (**E**) displays a representative analysis of (**C**). Blue to red represents the increase in event frequencies.

We analyzed the intracellular ATP concentrations to seek the cell viability loss mechanism. TMZ was used as a cytostatic control. Figure 4A,B indicates the cytotoxic effect of monotherapy with Osi and PKI-587 in the GBM95 and T98G cell lines, respectively. The combination of Osi and PKI-587 showed lower ATP concentrations, indicating the cytotoxic potential by blocking EGFR/PI3K/mTOR simultaneously. To confirm this cytotoxic effect, we performed a flow cytometry assay to identify annexin V-positive cells (indicative of apoptosis) after 24 h of monotherapy or polytherapy. Figure 4C,E shows a 2.57-fold increase in annexin V-positive GBM95 cells relative to the DMSO control. For the T98G cell line (Figure 4D), we see a 2.35-fold increase in annexin V-positive cells compared to the control. These results emphasize the benefits of blocking EGFR/PI3K/mTOR as a therapeutic alternative.

## 3. Discussion

Here we show that the combination of EGFR and PI3K/mTOR inhibitors displays synergy in GB suppression, considering EGFRvIII-positive or PI3K-β-positive cells. The combinations of PKI-587 with Osimertinib enhance their respective cytotoxic potencies and selectivity in monotherapies. The current therapeutic strategy available for GB presents significant limitations, particularly when considering the acquired resistance to TMZ.

Resistance to TMZ treatment has already been described extensively in GB [11]. Indeed, TMZ demonstrates low potency against various glioblastoma cell lines, which exhibit a resistant phenotype regardless of MGMT (T98G was the only tested cell line expressing MGMT). Regardless of the mechanism of resistance to TMZ, its existence represents a major problem for the chemotherapy treatment of GB in humans, allowing GB to be categorized as an unmet medical need.

While the wild-type EGFR is expressed by all the cell lines, the EGFRvIII mutation was found only in three of the five GB cell lines studied (GBM02, GBM03, and GBM95—Supplementary Figure S2). This leads to the constitutive activation of EGFR and posterior activation of the Akt/STAT pathways, bypassing the antitumoral effect of TMZ [7,12,13]. While the T98G cell line expresses MGMT, a common mechanism for TMZ resistance, the A172 cell line did not express EGFRvIII but expressed the PI3K p110β subunit. The p110β selectively converts phosphatidylinositol 4,5-bisphosphate into phosphatidylinositol 3,4,5-triphosphate, allowing the Akt anchorage to the plasmatic membrane, promoting the Akt phosphorylation and activation of mTOR, which will lead to GB cell survival and apoptosis inhibition. The G protein-coupled receptors bind and activate p110β selectively [14,15].

The effects of Osi are associated with its irreversible inhibition of the EGF receptor, leading to reduced activation of the PI3K/AKT/mTOR pathway. This inhibition results in decreased angiogenesis, cell migration, invasion, proliferation, and metabolism [16]. Furthermore, Osimertinib can irreversibly inhibit the EGFRvIII mutation, preventing constitutive pathway activation [17]. However, Osi did not show significant selectivity in the GBM02, GBM03, GBM95, T98G, and A172 cell lines. This low selectivity may be attributed to the inhibition of other pathways active in both tumor cells and non-tumor astrocytes, such as MNK1 and MNK2, part of the mitogen-activated protein kinase pathway. These kinases are associated with the synthesis of eukaryotic initiation factor 4G and the phosphorylation of eukaryotic translation initiation factor 4E [18].

The dual PI3K/mTOR inhibitor PKI-587 was selected for its promising in vitro potency against the α, β, γ, and δ isoforms of PI3K, as well as mTORC1 and mTORC2 [19]. PKI-587 also has established potency and safety in clinical trials involving solid and hematologic tumors, supporting its selection for this project [20].

We determined the cytotoxic potency of Osi and PKI-587 (Supplementary Figures S3 and S4) on the different tumor cell lines, observing a time-dependent effect (only for PKI) and the cytotoxic potency at 24 h and 72 h in the MTT test. Higher potency was observed at 72 h (Table 1). The cytotoxic selectivity index was determined, and it was shown that Osi is practically equipotent to inhibit the viability of tumor cells versus non-tumor human astrocytes. This was different from the behavior observed for PKI-587, which proved to be highly selective for the tumor cells studied (Table 1). Then, we studied the possibility of synergistic action obtained from the combination of selected concentrations of Osi and PKI-587 (Figure 2). We found that, indeed, the combination was able to improve cytotoxicity for GB cell lines, but not for the hASTR, suggesting potency and safety improvements.

The treatment with Osi hinders the closing of the wound in both tested cell lines (GBM95 and T98G), while the PKI-587 suppresses the migration in the GBM95, but not in the T98G. Its inactivity on T98G could be correlated to the expression of PI3K-β in the T98G lineage, which is absent in the GBM95 cell lines (Figure 1F). Our data show that Osi, when used in polytherapy, shows significant migration impairment in GBM95 and T98G cell lines. This may be associated with an important heterogenicity between different tumors with the same origin (for both cell lines and tumor patients). Nonetheless, polytherapy proved to be much more effective than monotherapy, and the combination of Osi with PKI-587 has great potential to inhibit the migration of GB, which is reflected in minimizing the metastatic potential of the tumor in vivo.

Tumor progression depends on the cell starting multiple cell cycles, reducing the regulation of each cell cycle checkpoint [21]. Several studies have highlighted the potential to suppress the cell cycle by inducing G0/G1 arrest in GB and other tumors, inhibiting the cell cycle pathways: EGFR/mitogen-activated protein kinase [22,23,24,25,26] and PI3K/mTOR [27,28,29,30,31]. Here we demonstrate that both monotherapy and polytherapy induce G0/G1 arrest in GBM95 (Figure 3E,F). The increase in G0/G1 cells is accompanied by a reduction in both S- and G2/M-phase cells, which suggests that the monotherapy and the polytherapy act by arresting the GB cells at the G1/S checkpoint.

The effect of Osimertinib as a cell proliferation inhibitor has already been described in the literature using an in vitro glioblastoma model. This occurs through the reduction of cyclin D1 expression and an increase in p21 expression, leading to cell cycle arrest in the G0/G1 phase [32]. A similar effect is observed with the PI3K inhibitor Paxalisib [32].

Although the PKI-587 alone inhibits proliferation, it does not inhibit the migration of the T98G cell line, which seems counterintuitive, but the PI3K/mTOR pathway has two distinct mechanisms that modulate cell proliferation and cell migration: (1) the hepatocyte growth factor receptor (c-MET)-dependent cell migration and (2) the Akt-dependent apoptosis-suppression and cell cycle inducing [33,34,35]. The suppression of c-MET reduces the migration and cell cycle arrest at G2/M phase, but not G1/S, in GB cells (e.g., T98G; [36]). Iezzi [37] used (in an ovarian tumor cell line) the combination of Akt pathway and c-MET inhibitors (PF-05212384 and crizotinib, respectively), showing a synergistic effect between both, corroborating that they act upon different pathways (Akt/c-MET), which explains the cell cycle arrest but not the migration inhibition of PKI-587 in the T98G cell line.

The ATP measurement classically allows the characterization of cytostatic or cytotoxic drugs [38], as cytotoxic drugs decrease ATP levels, while cytostatic drugs do not. Here we confirm that both drugs (Osi and PKI-587) are cytotoxic, and the combination maintains the cytotoxicity. The effect of cytotoxic drugs, reducing the establishment of the tumor, can only be matched, but not overcome, by cytostatic drug therapy [39]. Thus, the combination of Osi and PKI-587 has great potential to suppress the tumor progression, higher than the TMZ therapy (a cytostatic drug).

Cytotoxic drugs could display several adverse side effects like hyperuricemia (a feature of tumor lysis syndrome). This effect can be abrogated by differences in the mechanism of cytotoxicity [40,41]. Both monotherapy and polytherapy with Osi and PKI-587 increase the Anx V-positive cells, which indicates apoptosis or necrosis cell death. We can define the polytherapy mechanism of action as apoptosis-inducing therapy. Although PKI-587 did not show the capacity to induce apoptosis, its addition in the polytherapy increased the Osi capacity to induce apoptosis, showing one more layer of synergism. This synergistic effect on apoptosis, but not on necrosis, could minimize possible adverse side effects for Osi + PKI-587 therapy.

The high cytotoxicity of Osimertinib is already described in the literature [42] as being responsible for its unfavorable balance between antitumoral effects and adverse side effects. The proposed combination therapy may reduce the Osi dosage without decreasing its antitumoral effect but reduce the possibility of dose-dependent adverse effects. Using the combination of 5.5 μM of Osi and 0.0155 μM of PKI-587, we find a 92% killing in GBM95. It is worth mentioning that Osi in a monotherapy system only finds this killing potential in GBM95 cell lines at 50 μM (Supplementary Figure S3). So, applying the combination strategy, we were able to reduce the concentration of Osimertinib by around 10-fold. An improvement in the cytotoxic selectivity profile accompanied the improvement in the cytotoxic profile of the combination.

Taken together, our results disclose the synergistic effect of the combination of an irreversible EGFR inhibitor (Osimertinib) and a dual PI3K and mTOR inhibitor (Gedatolisib or PKI-587) on human GB cell lines. This combination, compared to monotherapy treatment, resulted in a cytotoxic response improvement, and a reduction in cell migration, apoptosis induction, and the selectivity index. These data support the hypothesis that such a combination may have favorable results in animal models, encouraging future in vivo preclinical trials with this combination.

## 4. Methods

### 4.1. Ethics Statement and GB Cell Cultures

This study was approved by the Ethics Committee at the Center for Health Sciences in the Federal University of Rio de Janeiro and by the Brazilian Ministry of Health Ethics Committee (CONEP Protocol No. 2340). All experiments were performed according to relevant guidelines and regulations. The GBM02, GBM03, and GBM95 GB cell lines were established and characterized in our laboratory, as previously described [43]. The T98G cell line originated from ATCC. A172 GB cell line was purchased from ECACC. All the GB cells were maintained in Dulbecco’s modified Eagle’s medium (DMEM), high glucose, supplemented with 10% fetal calf serum (FCS) at 37 °C and in a controlled atmosphere containing 5% CO_2_. The adult human astrocyte cells (hASTR) were isolated from patients selected for surgical treatment of temporal lobe epilepsy associated with hippocampus sclerosis at the Hospital Universitario Clementino Fraga Filho. Human astrocytes were characterized morphologically by expressing typical astrocyte markers (human leukocyte antigen (HLA), GFAP, and glutamate-aspartate transporter. All patients provided written informed consent to participate in the study, and the procedures were agreed to by the Brazilian Ministry of Health Ethics Committee (CONEP Protocol No. 2340).

### 4.2. MTT Assay

Metabolically active cells were assessed using the 3-(4,5-dimethylthiazol-2-yl)-2,5-diphenyl tetrazolium bromide (MTT) reduction colorimetric assay, as described by Balça-Silva [44]. GB cells were plated (4 × 10^5^ cells/mL for GBM02, GBM03, and GBM95, and 5 × 10^5^ cells/mL for A172 and T98G in the 24 h group; 2 × 10^5^ cells/mL for GBM02, GBM03, and GBM95, and 5 × 10^5^ cells/mL for A172 and T98G in the 72 h group) in 96 multi-well plates and then were incubated manually with TMZ (10/50/100/500 μM), PKI-587 (50/10/1/0.1/0.01/0.001 mM for 24 h or 72 h), Osimertinib (50/25/10/5/1/0.1 mM for 24 h, and 50/10/1/0.1/0.01 mM for 72 h). All drugs were reconstituted in DMSO and diluted in sterile water at a final 2% DMSO concentration. Water + 2% DMSO was used as a control. The absorbance was read in a microplate reader at 570 nm, and the CC_50_ was calculated using the Prism 7 statistical software (GraphPad Software, San Diego, CA, USA). The synergistic effect between Osi and PKI-587 was based on the MTT assay, considering the Chou–Talalay method, based on the median-effect equation [10]. We obtained combination curves for Osimerinib and PKI-587 as single agents and in constant ratios of their potency values (0.25×, 0.5×, 1×, 2×, and 4× CC_50_). Results of drug combinations were obtained by CompuSyn version 1.0 (ComboSyn, Paramus, NJ, USA), and expressed in a combination index (CI) versus fraction affected (Fa) graph. Combinations with CI values lower than one are considered synergy, 1 < CI < 1.5 is considered additivity, and antagonism is CI > 1.5 [10]. The selectivity index was determined following Chao [45], dividing the CC_50_ of the selected drug in the GB cell line by the CC_50_ of the same drug in a human astrocyte cell.

### 4.3. Western Blot Analysis

Protein expressions were analyzed using Western blot analysis, as described by Towbin [46], and after modifications by Balça-Silva [44]. The primary antibody dilutions were EGFRvIII (1:1000, CellSignaling, Danvers, MA, USA), PI3K p110β (1:1000, CellSignaling), MGMT (1:1000, CellSignaling), Vimentin (1:250, Invitrogen, Waltham, MA, USA), GAPDH (1:1000, Novus Biologicals, Centennial, CO, USA). The immunocomplexes were detected with an anti-rabbit antibody (1:10,000, Invitrogen) and anti-mouse antibody (1:10,000, Invitrogen) conjugated with horseradish peroxidase. Bands were obtained after exposing the membranes to the Universal Hood III photo documenter (BioRad, Contra Costa County, CA, USA) and incubating the membranes in SuperSignal WestFemto (ThermoScientific, Waltham, MA, USA). Bands were analyzed through densitometry scanning, and GAPDH expression was used as a loading control.

### 4.4. Cell Migration by Scratch Wound Assay

Cell migration was performed according to the method described by Liang [47]. The GBM cell monolayer was scraped in a straight line with a p200 pipette tip. The debris was removed by washing the cells with PBS. Then, DMEM (without FCS) supplemented with 10 μM Ara-C (for GBM95) or DMEM supplemented with 2.5% FCS and 10 μM Ara-C (for T98G) was added to the well. Ara-C was used as a proliferation inhibitor. Then, 5.5 μM of Osi and 0.0155 μM of PKI-587 were added to the GBM95 cell line, alone or combined. For the T98G cell line, the selected concentration was 13 μM of Osi and 0.186 μM of PKI-587. ImageJ software (Version 1.8.0; National Institutes of Health) was used to record the coordinates for each scratch location using a “wound healing size tool” plugin [48]. The scratch width at 24 h was compared to the original (0 h).

### 4.5. Cell Cycle Analysis

The cell cycle was analyzed by PI incorporation [49]. Briefly, GBM95 cell lines were seeded at a density of 2 × 10^6^ cells/well in a 24-well plate and incubated for 24 h with 5.5μM of Osi and 0.0155 μM of PKI-587, alone or combined. Then, the cells were enzymatically detached (0.25% trypsin in DMEM), and 2 × 10^6^ cells were pelleted down (5 min/500× *g*) and resuspended in 3 mL of cold 70% ethanol, dropwise. After 1 h at 4 °C, the cells were centrifuged and washed with PBS twice (10 min/700× *g*). To the final pellet, 50 µg of PI and 1 ug RNAse A were added to PBS (1 mL final volume) and incubated for 4 h at 4 °C. The PI incorporation was measured in the CytoflexS (Beckman Coulter, Brea, CA, USA) cytometer.

### 4.6. ATP Quantification

ATP measurement was performed using luminescent ATP detection (Abcam 113849, Cambridge, UK) following the manufacturer’s instructions. Cells and isolated or combined drugs were incubated for 72 h at the following combinations: GBM95 (5.5 μM Osi and 0.0155 μM PKI-587) and T98G (13 μM Osi and 0.186 μM PKI-587). TMZ (500 μM) was used as a control.

### 4.7. Apoptosis Assay

To evaluate apoptosis, 2 × 10^6^ cells were seeded and incubated for 24 h. Then, 5.5 μM of Osi and 0.0155 μM of PKI-587 were added to the GBM95 cell line. The selected combination for the T98G cell line was 13 μM of Osi and 0.186 μM of PKI-587. For both cells, the drugs were administrated alone or in combination. After 24 h, 1 × 10^6^ cells were pelleted down (5 min/500×g) and resuspended in 100 µL of binding buffer 1×. Next, 5 µL of Annexin V (AnxV) and 5 µL of propidium iodide (PI) were added to the cell suspension, and the incubation was performed at room temperature for 15 min in the dark [50]. Then, we added 400 µL of binding buffer 1× and proceeded to the CytoflexS (Beckman Coulter) cytometer analysis.

### 4.8. Statistical Analysis

Data (presented as mean + SEM throughout) were analyzed, and multiple comparisons were made by ANOVA using Bonferroni’s corrections with Windows-supported SigmaPlot version 11.0 (Erkrath, Germany). Pairwise comparisons were done with two-tailed t-tests (separate variances). *p* < 0.05 was considered significant. The CI values were calculated using CompuSyn version 1.0. All data shown are representative of three independent experiments. Potency values were calculated using the Prism 7 statistical software (GraphPad Software, San Diego, CA, USA).

## Figures and Tables

**Table 1:** Cytotoxic potency of Osimertinib (Osi) and PKI-587, measured by their concentration of cytotoxicity 50% (CC_50_) in a 24 h and 72 h MTT assay.

	**24 h**		**72 h**		**72 h**
**Osimertinib**	**Emax (%)**	**CC_50_ (µM)**	**Emax (%)**	**CC_50_ (µM)**	**SI**
GBM02	99.3	13.08 (3.8–17.1)	98.3	13.1 (10.1–19.2)	1.03
GBM03	99.2	11.54 (9.6–14.2)	99	6.6 (5.4–8.5)	2.04
GBM95	99.5	10.4 (7.4–13.12)	98.2	11.0 (8.4–13.7)	1.22
T98G	97.9	9.9 (8.8–10.98)	97.9	11.06 (8.3–14.1)	1.22
A172	99.1	29 (26.1–35.2)	99.5	11.83 (9.6–17.2)	1.14
hASTR	-	-	99.7	13.49	
	**24 h**		**72 h**		**72 h**
**PKI-587**	**Emax (%)**	**CC_50_ (µM)**	**Emax (%)**	**CC_50_ (µM)**	**SI**
GBM02	57.7	54.2 (22.1–534.7)	96.7	0.14 (0.03–0.6)	200
GBM03	87.4	23.1 (14.6–38.35)	95.5	0.085 (0.02–0.4)	329.4
GBM95	82.2	2.1 (0.63–8.4)	99.7	0.03 (0.01–0.1)	933.3
T98G	98.4	5.1 (1.45–14.34)	96.8	0.20 (0.11–0.4)	140
A172	92.4	2.8 (0.53–14.22)	98.5	0.55 (0.2–1.5)	51
hASTR	-	-	53	28	

Optic density was measured at 570 nm, and data were normalized by the non-treated control (usual media +1%DMSO). CC50 and Emax were calculated by concentration–response curves for the target compounds in all GB cells after 24 and 72 h of incubation. Values are the means and ranges of three independent experiments in triplicate. SI = selectivity index.

## Data Availability

The datasets generated during and analyzed during the current study are available from the corresponding author upon reasonable request. All data generated or analyzed during this study are included in this published article (and its Supplementary Information files).

## Supplementary Materials

The following supporting information can be downloaded at: https://www.mdpi.com/article/10.3390/ph17121623/s1, Supplementary Figure S1—related to Figure 1. The original membranes of the two experiments are displayed in Figure 1. Immunoblot showing the expression of PI3K β, GAPDH, MGMT, vimentin, and EGFRvIII. Supplementary Figure S2—related to Figure 1. Immunofluorescence of EGFRwt and EGFRvIII expression in all five glioblastoma lineages. Supplementary Figure S3—related to Table 1. Osimertinib potency and efficacy at 24 h and 72 h against GB cell lines and human astrocytes. Supplementary Figure S4—related to Table 1. PKI-587 potency and efficacy at 24 h and 72 h against GB cell lines and human astrocytes.

## Abbreviations

Akt	Protein kinase B
AnxV	Annexin V
BBB	Brain–blood barrier
c-MET	Hepatocyte growth factor receptor
CC50	Concentration of cytotoxicity of 50%
CI	Combination index
CNS	Central nervous system
EGFR	Epidermal growth factor receptor
Emax	Maximum effect
Fa	Fraction affected
FCS	Fetal calf serum
GB	Glioblastoma
hASTR	Human astrocyte
MGMT	O6-methylguanine-DNA methyl transferase
mTOR	Mammalian target of rapamycin
Osi	Osimertinib
PI	Propidium iodide
PI3K	Phosphoinositide 3-kinase
PKI-587	Gedatolisib
PTEN	Phosphatase and tensin homologue
SI	Selectivity index
TMZ	Temozolomide
