# Supplementary material for: Repurposing Osimertinib and Gedatolisib for Glioblastoma Treatment: Evidence of Synergistic Effects in an In Vitro Phenotypic Study
Source: Pharmaceuticals (Basel). 2024 Dec 3;17(12):1623. doi: 10.3390/ph17121623 (PMC11678499; doi:10.3390/ph17121623)
Supplement: Supplementary file 1 [file pharmaceuticals-17-01623-s001.zip › pharmaceuticals-3312342-supplementary.pdf]

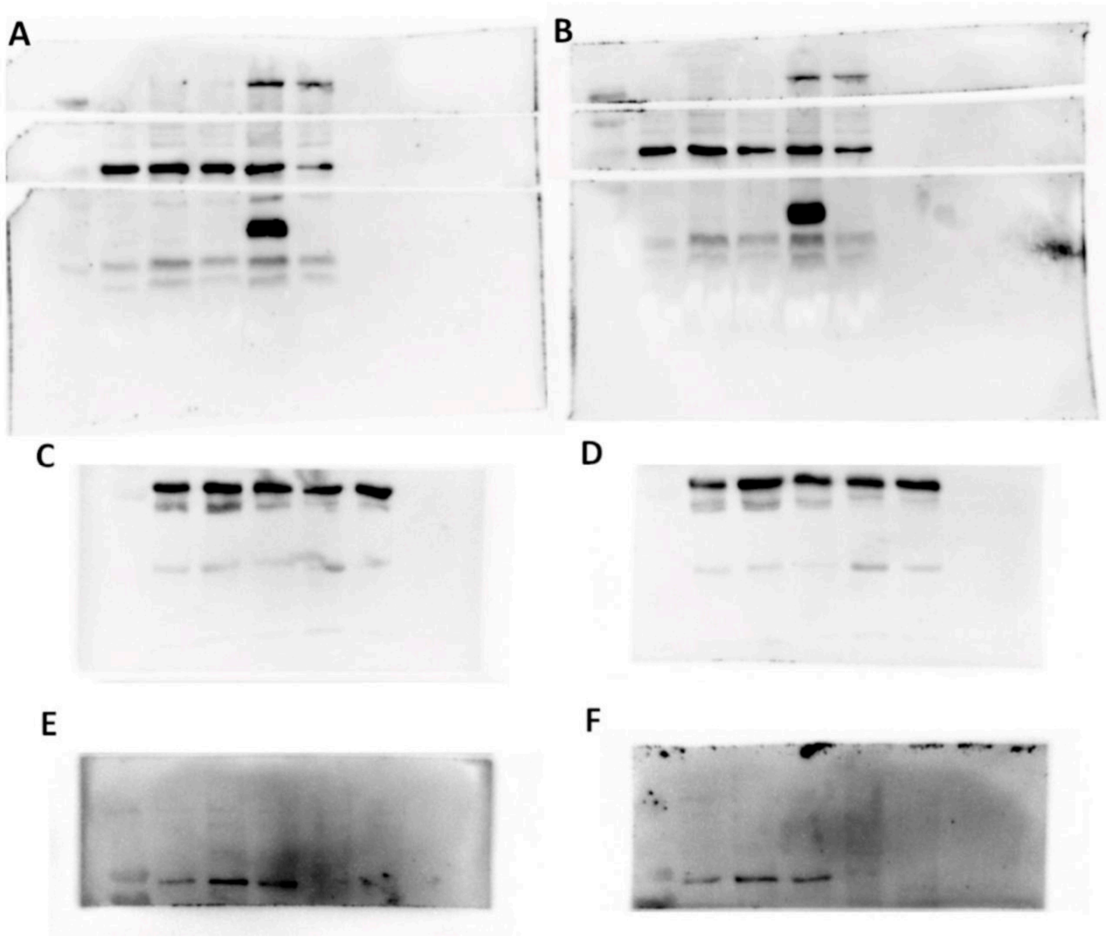

Supplementary Figure S1 – Related to Figure 1. The original membranes of the two experiments are displayed in Figure 1. Immunoblot showing the expression of PI3K β (110 KDa), GAPDH (37 KDa), and MGMT (21KDa) (**A and B);** Vimentin (57KDa) (**C and D**); and EGFRvIII (130 KDa) (**E and F**). Lane 1: Ladder, Lane 2-6: GBM02, GBM03, GBM95, T98G, A172 in all immunoblots.

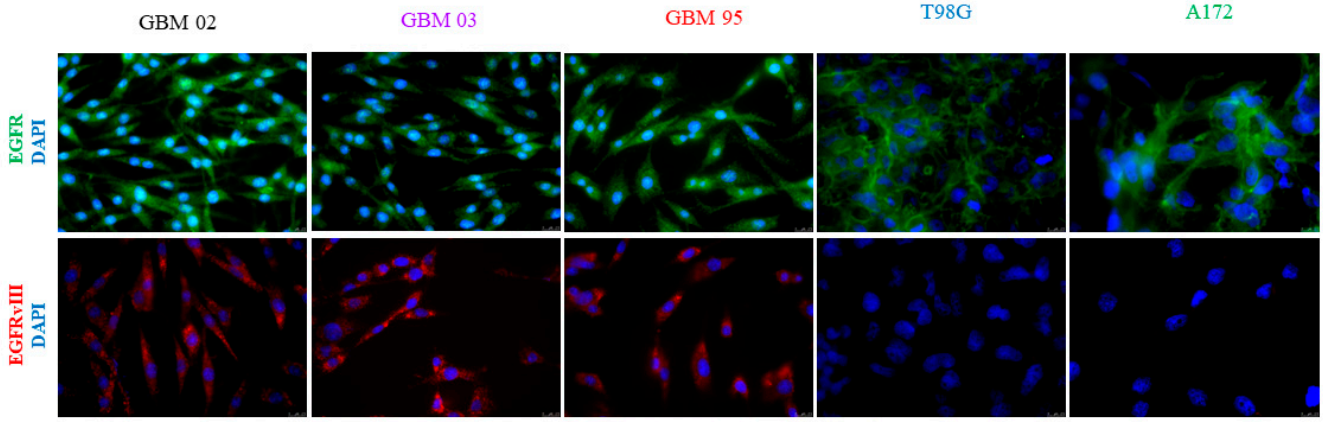

Supplementary Figure S2 – Related to Figure 1. The complementary results of EGFRwt and EGFRvIII are in Figure 1. Immunophenotypes of GB lineages, show the expression of EGFRwt (green stained), in all five GB lineages, and EGFRvIII (red stained), in GBM02, GBM03, and GBM95. Nuclei were stained in blue (DAPI)—magnification of 630x for all images.

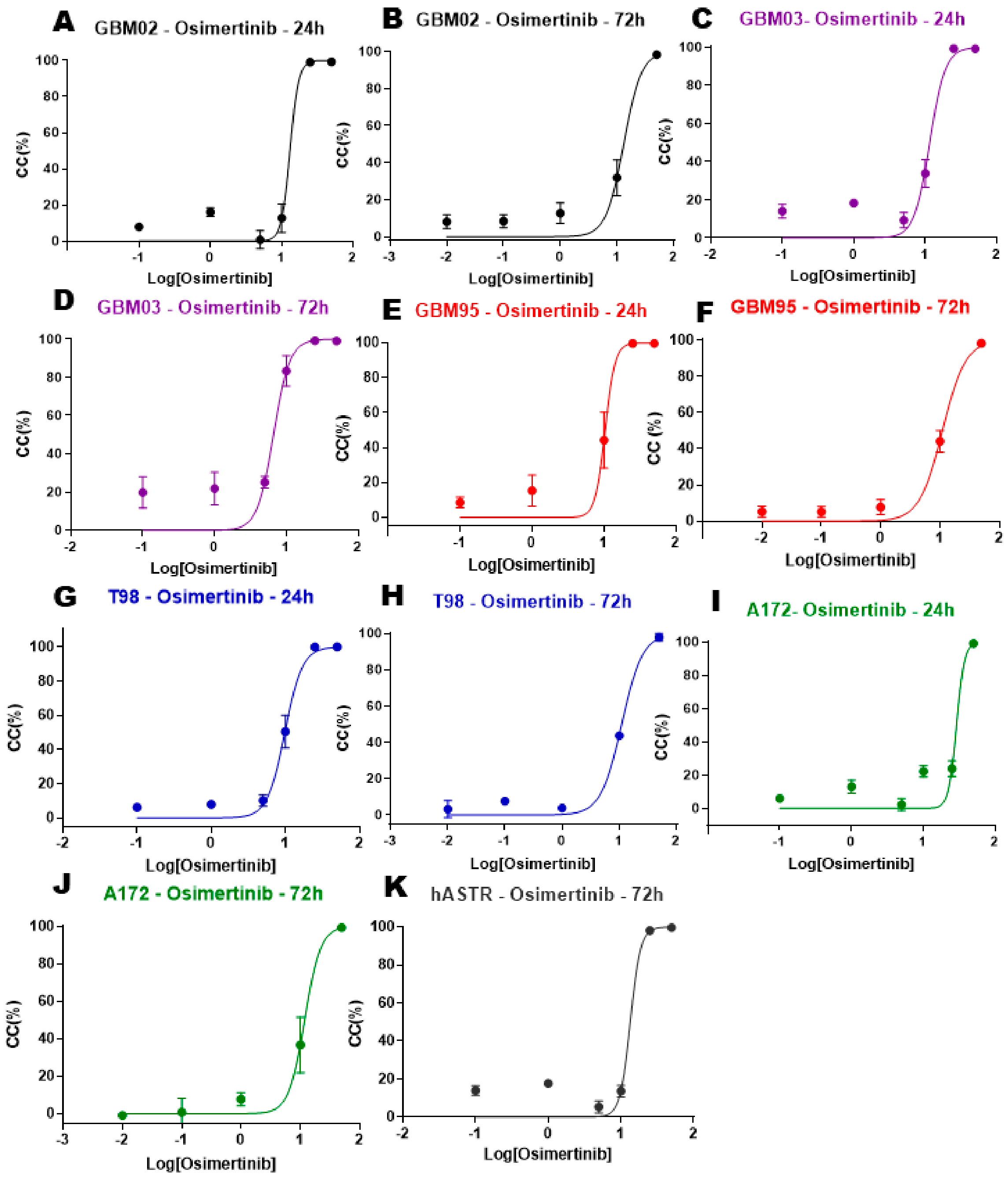

Supplementary Figure S3 – Related to Table 1. Potency and efficacy of Osimertinib at 24h and 72h against GBM02 (A and B), GBM03 (C and D), GBM95 (E and F), T98G (G and H), A172 (I and J) GB lineages and human astrocytes (K). Values are means ± SEM of three independent experiments in triplicate.

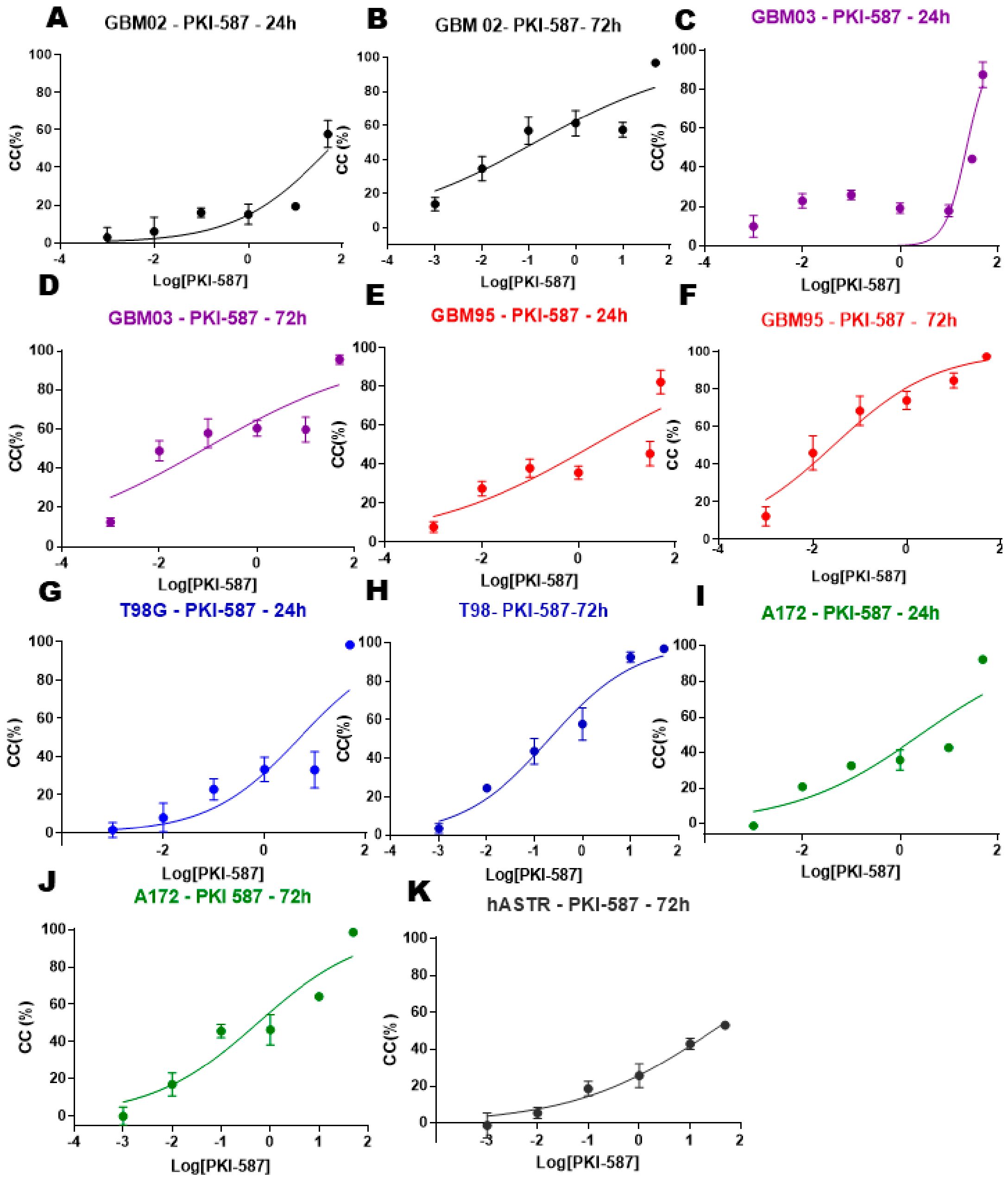

Supplementary Figure S4 – Related to Table 1. Potency and efficacy of PKI-587 at 24h and 72h against GBM02 (A and B), GBM03 (C and D), GBM95 (E and F), T98G (G and H), A172 (I and J) GB lineages and human astrocytes (K). Values are means ± SEM of three independent experiments in triplicate.
